# *Helicobacter pylori vacA* transcription is genetically-determined and stratifies the level of human gastric inflammation and atrophy

**DOI:** 10.1136/jclinpath-2016-203641

**Published:** 2016-05-17

**Authors:** Charlotte G Sinnett, Darren P Letley, Geetha L Narayanan, Sapna R Patel, Nawfal R Hussein, Abed M Zaitoun, Karen Robinson, John C Atherton

**Affiliations:** 1 NIHR Biomedical Research Unit in Gastrointestinal and Liver Diseases at Nottingham University Hospitals NHS Trust and the University of Nottingham, School of Medicine, University of Nottingham, Nottingham, UK; 2 Department of Cellular Pathology, Nottingham University Hospitals NHS Trust, Nottingham, UK

**Keywords:** TOXIN, MICROBIAL PATHOGENIC, HELICOBACTER PYLORI, INFLAMMATION, GASTRIC PATHOLOGY

## Abstract

**Aims:**

*Helicobacter pylori* infection is the major cause of peptic ulceration and gastric cancer, and an important virulence determinant is its vacuolating cytotoxin *vacA.* Previously, we have described allelic variation in *vacA* which determines toxin activity and disease risk. Here we aimed to quantify *vacA* mRNA expression in the human stomach, define its genetic determinants and assess how well it predicts gastric pathology.

**Methods:**

Gastric biopsies were donated by 39 patients with *H. pylori* infection attending for endoscopy at Queen's Medical Centre, Nottingham, UK. Total RNA was extracted, and *vacA* mRNA quantified by reverse transcriptase quantitative PCR. Separate biopsies were histologically scored for inflammation and atrophy using the updated Sydney system. *H. pylori* strains were isolated from further biopsies, and the nucleotide sequence upstream of *vacA* determined.

**Results:**

*vacA* mRNA levels in human stomachs varied by two orders of magnitude independently of *vacA* allelic type. Among *vacA* i1-type (toxic) strains, increased *vacA* expression was strongly associated with higher grade gastric inflammation (p<0.02), neutrophil infiltration (p<0.005) and the presence of atrophy (p<0.01). A polymorphism at nucleotide +28 near the base of a potential stem-loop structure within the 5′ untranslated region was significantly associated with *vacA* transcript level and inflammation.

**Conclusions:**

Increased gastric *vacA* expression during *H. pylori* infection is associated with inflammation and premalignant pathology. The +28 nucleotide within the *vacA* 5′ stem-loop stratifies disease risk among toxic *vacA* i1-type strains.

## Introduction

*Helicobacter pylori* persistently colonises the stomach of approximately half the world's population causing chronic gastritis, which usually remains asymptomatic. However, some individuals develop overt disease, such that *H. pylori* is the leading cause of peptic ulcer disease (PUD), mucosa-associated lymphoid tissue (MALT) lymphoma and distal gastric adenocarcinoma (GC).^1^ Given the prevalence of infection, *H. pylori*-related gastric diseases are a major global health burden. In 2010, the estimated global deaths from PUD and GC were 0.25 million and 0.75 million, respectively.^2^ GC is estimated to be the third biggest cause of cancer-related death worldwide.^3^ Determinants of disease risk for a specific *H. pylori*-infected individual include: the virulence of the infecting strain, host factors such as gene polymorphisms and immune response, and environmental factors such as diet and smoking.^1^

Two important *H. pylori* virulence factors strongly associated with gastric and duodenal disease are the cytotoxin associated gene A (*cagA*) and the vacuolating cytotoxin, VacA. The CagA protein is translocated into host cells by a type IV secretion system encoded on the *cag* pathogenicity island that is present in about 70% of strains. Here it becomes phosphorylated by Src-family kinases and interacts with several host signalling factors, such as the proto-oncogene SHP2, modulating important cancer-related signalling pathways leading to increased cell proliferation and motility, as well as the expression of pro-inflammatory cytokines via the activation of nuclear factor kappa-light-chain-enhancer of activated B cells (NF-κB).^4^ Transgenic mice expressing CagA protein develop gastrointestinal and haematopoietic neoplasms, directly demonstrating the oncogenic properties of this important virulence determinant.^5^ VacA has many cellular effects in vitro, including: inducing vacuolation of epithelial cells, causing mitochondrial damage leading to apoptosis; reducing transepithelial resistance; inhibiting T cell proliferation; and activating mitogen-activated protein (MAP) kinase pathways.^6^ It also causes epithelial damage when given orally to mice.^7^ Many of these effects depend on VacA forming anion-selective channels in cell membranes.^6^

The *vacA* gene is present in all *H. pylori* strains, and virtually all produce VacA protein; however, not all are fully cytotoxic. While *vacA* is generally well conserved, three regions with significant allelic diversity exist: the signal region (s1/s2), the mid-region (m1/m2) and the intermediate region (i1/i2).^8^ ^9^ Mosaicism occurs such that all forms of the toxin exist, although some alleles are rare. Functionally, the signal and intermediate regions determine toxin activity: s1i1 forms are fully active while s1i2 and s2i2 forms are less so. The vacuolating activity of s2 forms is blocked by an N-terminal extension to the mature toxin.^10–12^ Mutagenesis studies show that the intermediate region is a direct determinant of toxin activity, but the underlying mechanism remains undetermined.^9^

In Western Europe and the USA, where *vacA* allelic diversity is common, s1m1 strains are associated with more gastric inflammation, PUD and GC.^6^ Recently, the *vacA* i1 genotype has been shown to be an even better marker of gastric disease risk, as expected given that it is a direct determinant of toxicity.^9^ ^13^ However, these associations are not absolute, and in some populations, particularly in East Asia, most *H. pylori* strains are *vacA* i1-type, making the intermediate region an unsuitable disease marker in such areas.^14^ ^15^

*vacA* transcription varies between *H. pylori* strains grown in vitro.^16^ ^17^ We hypothesised that the amount of VacA produced during infection, as well as its activity, would have important implications for disease. We aimed to quantify *vacA* transcript levels in gastric biopsies from patients with *H. pylori* infection, correlate this with inflammation, atrophy and intestinal metaplasia, and identify genetic determinants of *vacA* expression which may be additional markers of disease risk.

## Materials and methods

### Bacterial strains

*H. pylori* strains were grown on blood agar base 2 plates supplemented with 5% horse blood (Oxoid, Basingstoke, UK) for 24–48 h at 37°C under humid, microaerobic conditions in a MACS-VA500 workstation (Don Whitley Scientific, Shipley, UK)*.* Clinical *H. pylori* isolates were genotyped for *vacA* allelic type and *cagA* status by PCR as previously described.^9^

### Patient samples

Corporal gastric biopsies were donated by 39 patients with *H. pylori* infection (median age 56 years, range 30–82 years; 51% male) attending the Queen's Medical Centre, Nottingham, UK, for routine upper gastrointestinal endoscopy for dyspeptic symptoms. Informed written consent and approval from the Nottingham Research Ethics Committee 2 (08/H0408/195) was obtained. Patients taking proton pump inhibitors, non-steroidal anti-inflammatory drugs (NSAIDs), >150 mg/day of aspirin or antibiotics 2 weeks preceding endoscopy were excluded. Biopsies for isolation of *H. pylori* were immediately placed into isosensitest broth (Oxoid) containing 15% (v/v) glycerol, and then swabbed onto blood agar plates and cultured. For histology, formalin-fixed corporal biopsies (two per patient to avoid patchy changes) were paraffin-embedded, sectioned and stained with H&E for assessment of inflammation and atrophy. Grading was performed using updated Sydney scoring^18^ (0=not present, 1=mild, 2=moderate and 3=substantial) by an experienced histopathologist (AMZ) blinded to other data. Histological scores were representative of both corporal biopsies examined for each patient. A single corporal biopsy for each patient was taken for RNA analysis and immediately preserved in RNAlater (Sigma-Aldrich, Poole, UK).

### Nucleotide sequencing of the *vacA*–*cysS* intergenic region

Genomic DNA was extracted from clinical *H. pylori* isolates as previously described.^19^ The *vacA* sequence from nucleotides 520–1055 (GenBank U05676), including the intergenic region between *vacA* and the upstream gene *cysS* (cysteinyl-tRNA synthetase), was PCR-amplified using primers DL1^20^ and VA1-R,^8^ and the product sequenced by the Biopolymer Synthesis and Analysis Unit, University of Nottingham. Sequences were aligned using MegAlign (DNAStar, Madison, Wisconsin, USA). Nucleotide positions where three or more strains differed to the consensus were defined as polymorphic.

### Reverse transcriptase quantitative PCR

Total RNA was purified from gastric biopsies using rotor-stator homogenisation and an RNeasy Mini Kit (Qiagen, Manchester, UK). Residual genomic DNA was removed by DNA-*free* DNase treatment (Life Technologies, Paisley, UK). cDNA was reverse transcribed from 200 ng RNA using an Omniscript reverse transcriptase kit (Qiagen) and random hexamer primers (GE Healthcare Life Sciences, Little Chalfont, UK). For each sample, negative control reactions were prepared without reverse transcriptase to confirm the absence of genomic DNA. Triplicate reverse transcriptase quantitative PCRs (RT-qPCRs) were performed using a Quantitect SYBR Green PCR kit and a Rotor-Gene 3000 real-time PCR system (both Qiagen). *vacA* mRNA levels were calculated relative to a comparator biopsy cDNA sample included in all experiments (arbitrarily assigned an expression level of 100) using the Pfaffl method,^21^ normalising to the *H. pylori* reference gene *16S rRNA* to correct for differences in *H. pylori* density or sample amount. A 259 bp *vacA* amplicon was amplified using primers VA1-F and VA1-R,^8^ and a 164 bp *16S rRNA* amplicon amplified by primers 16S-rRNA-F (5′ CGATGAAGCTTCTAGCTTGC 3′) and 16S-rRNA-R (5′ ATAGGACATAGGCTGATCTC 3′). Thermocycling conditions were: 95°C for 15 min followed by 35 cycles of 95°C for 30 s, 56°C for 60 s and 72°C for 30 s. No template controls were included and melt curve analysis confirmed amplification specificity. Primer efficiencies, calculated from serial dilutions of the comparator sample, were 0.91 (R^2^=0.98) and 0.57 (R^2^=0.99) for *vacA* and *16S rRNA*, respectively.

### Statistical analysis

Associations between gastric *vacA* mRNA data and genotype, histological data, disease type and promoter region polymorphisms were evaluated by Mann–Whitney U tests or by Kruskal–Wallis analysis of variance (ANOVA) for multiple comparisons, with multiplicity adjusted pairwise p values calculated by Dunn's test. Tests were performed using GraphPad Prism 6 software, and two-tailed p values less than 0.05 were taken as being statistically significant.

## Results

### *H. pylori vacA* expression in the human stomach varies considerably between strains

While *vacA* allelic type is a major determinant of gastric cancer risk among patients with *H. pylori* infection, the association is imperfect, and we hypothesised that the amount of VacA produced by a strain may also be important. Reliable quantification of VacA production by *H. pylori* in the human stomach is hampered by the lack of specific antibodies, so we aimed to assess levels of *vacA* mRNA in gastric biopsies using RT-qPCR. We quantified *vacA* mRNA in corporal gastric biopsies from 39 patients with *H. pylori* infection. *vacA* transcripts were detected in biopsies from 21 patients, with levels varying from 0.1 to 46.8 relative *vacA* mRNA units. For the remaining 18 patients, no *vacA* expression was detected in vivo. By assaying serially diluted comparator sample cDNA, we determined our *vacA* mRNA detection limit as being eightfold below the lowest positive value obtained from patient biopsies. Thus strains with undetectable *vacA* mRNA have expression below this level.

### The level of *vacA* expression in vivo does not correlate with *vacA* type

We determined the *vacA* allelic type of each strain by PCR^8^ ^9^ ^22^ to assess whether this correlated with gastric *vacA* mRNA level. Two patients were excluded because typing suggested the presence of multiple strains with different *vacA* types, leaving 37 patients in our analysis. There was no significant difference in relative *vacA* mRNA levels between toxic i1-type and non-toxic i2-type strains (medians 0.93 (n=27) and 0.96 (n=10), respectively, p=0.99, Mann–Whitney U test). There were only four patients infected with s2-type strains, too few to compare signal region types; however, one was a high- and two were low-level transcribers. Moreover, one had undetectable *vacA* mRNA. We also assessed *cagA* status by PCR for all 39 infecting strains, and again there was no association with relative gastric *vacA* mRNA level (medians 0.93 for *cagA*+ strains (n=29) and 0.96 for *cagA*- strains (n=10), p=0.47).

### For toxic *vacA* i1-type strains, *vacA* mRNA levels in the human stomach are closely associated with gastric inflammation and precancerous change

Next, we assessed whether *H. pylori vacA* expression in the stomach was associated with inflammation and precancerous damage by scoring histological parameters using the updated Sydney system.^18^ We previously showed that only i1-type strains are actively toxic in vitro.^9^ In agreement with this, 8 of the 10 patients infected with i2-type strains had mild (grade 1) inflammation and none had atrophy or intestinal metaplasia. Our main interest was why i1-type strains have different associations with inflammation and damage; so we limited subsequent analysis to 25 patients infected with i1-type strains for which histology samples could be located.

**Figure 1:**
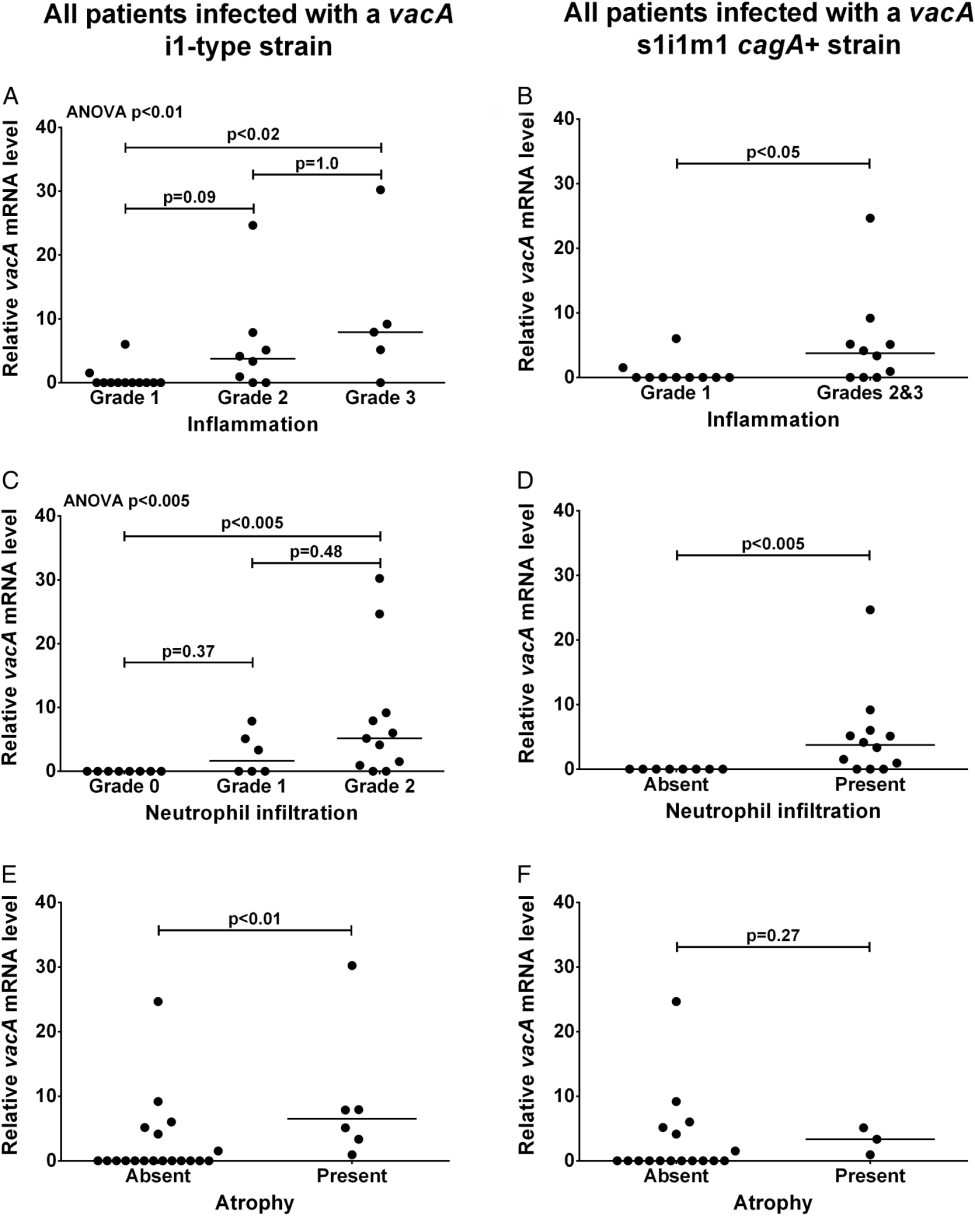
*vacA* mRNA level in the human stomach infected with *Helicobacter pylori* is associated with inflammation and atrophy. Gastric biopsies were scored for inflammation (A and B), neutrophil infiltration (C and D) and atrophy (E and F) using the updated Sydney system by a qualified histopathologist blind to all data. Relative *vacA* mRNA levels in separate gastric biopsies were quantified by reverse transcriptase quantitative PCRs using the Pfaffl method^21^ with *16S rRNA* as a reference gene (arbitrary units). Data were stratified by *vacA* i1-type (A, C and E; n=25) or *vacA* s1i1m1, *cagA*^+^ genotype (B, D and F; n=20). Multiple comparisons for inflammation and neutrophil infiltration grades (A and C) were assessed by Kruskal–Wallis analysis of variance (ANOVA) and multiplicity adjusted pairwise p values calculated by Dunn's test are shown. Pairwise comparisons (B and D–F) were assessed by Mann–Whitney U tests for statistical significance.

First we assessed the association between *vacA* expression and ‘chronic inflammation’ score, defined by the density of mononuclear inflammatory cells in the biopsy specimen. Median relative *vacA* mRNA level was significantly higher in patients with grade 3 inflammation than those with grade 1 inflammation (p<0.02; figure 1A). Patients with grade 2 inflammation had intermediate *vacA* mRNA levels. Seventeen patients had grade 1 or 2 neutrophil infiltration, referred to as ‘activity’ in the Sydney system, eight had no activity (grade 0) and none had grade 3 activity. Median relative *vacA* mRNA level for patients with grade 2 activity was significantly higher than for patients with no neutrophil infiltration (grade 0; p<0.005); patients with grade 1 activity were intermediate (figure 1C). Only six patients had gastric atrophy, all grade 1. Median relative *vacA* mRNA level was significantly higher in patients with atrophy than without (p<0.01; figure 1E). Only two patients had intestinal metaplasia, insufficient for statistical analysis; however, their median relative *vacA* level was 6.5 compared with 0.0 for the patients without intestinal metaplasia.

To avoid confounding effects from differences in *vacA* signal and mid-region type, and *cagA* status, we next stratified our data based on the 20 patients infected with *vacA* s1i1m1 *cagA*+ strains. We combined data for inflammation grades 2 and 3 to avoid small group sizes. Median relative *vacA* mRNA level was significantly higher for patients with grade 2 or 3 inflammation than grade 1 (p<0.05; figure 1B), and for patients with neutrophil infiltration than for those without (p<0.005; figure 1D). Only three patients infected with *vacA* s1i1m1 *cagA*+ strains had atrophy, too few for reliable statistical analysis (figure 1F).

### The level of *vacA* transcription in vivo does not correlate with PUD

**Figure 2:**
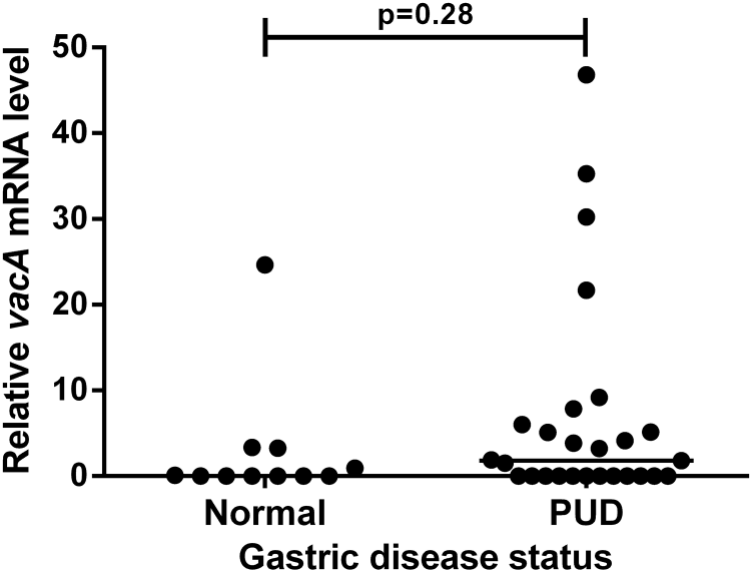
Peptic ulcer disease (PUD) does not correlate with in vivo *vacA* mRNA level. Pathological findings were recorded by a qualified gastroenterologist during upper gastrointestinal endoscopy for dyspeptic symptoms. Patients with past or present gastric or duodenal ulcers or erosions were defined as a PUD group. Patients showing no pathology were defined as normal. Patients taking proton pump inhibitors or antibiotics in the 2 weeks preceding endoscopy were excluded from the study. Relative *vacA* mRNA levels in gastric biopsies were quantified by reverse transcriptase quantitative PCRs with *16S rRNA* as a reference gene (arbitrary units). Data were assessed by Mann–Whitney U tests for statistical significance.

Of the 39 patients studied, 27 had past or present gastric or duodenal ulcers or erosions and were collectively defined as a PUD group. Only one patient had GC and was omitted from this analysis. The remaining 11 patients showed no pathology on endoscopy and were defined as a normal group. There was no significant difference in median relative *vacA* mRNA level between the PUD and normal groups (figure 2). However, our study was not powered to detect such a difference. There remained no significant difference in median relative *vacA* mRNA levels when comparing the 22 patients with active ulcers and the normal group (medians 0.76 and 0.0, p=0.50).

### In vivo *vacA* mRNA levels are not associated with promoter polymorphisms, but are associated with a polymorphism in a potential 5′ stem-loop in the *vacA* transcript

Having shown that inflammation and premalignant disease were associated with increased *vacA* mRNA among toxigenic, i1-type strains, we considered it important to assess the genetic determinants of high level *vacA* expression in vivo. We compared the nucleotide sequence between *vacA* and the upstream gene *cysS* for 27 strains to identify polymorphisms that correlated with our gastric *vacA* mRNA data (see online supplementary figure S1). Interestingly, 31/110 nucleotides in the *vacA* promoter region (end of *cysS* to *vacA* transcriptional start site) were polymorphic compared with just 11/119 nucleotides in the 5′ untranslated region (5′ UTR) of the encoded transcript (p=0.0003, Fisher's exact test). In agreement with previous studies,^17^ ^23^ the *vacA* promoter had three polymorphisms: G/A/T(−32) within the −35 motif; and G/T(−14) and G/T(−7) within the extended −10 motif. Four polymorphisms were observed within a previously identified imperfect inverted repeat upstream of the −35 motif,^17^ with three strains having a perfect inverted repeat. None of these, or the remaining polymorphisms within the promoter region, correlated with gastric *vacA* mRNA level.

10.1136/jclinpath-2016-203641.supp1 Supplementary figure

**Figure 3:**
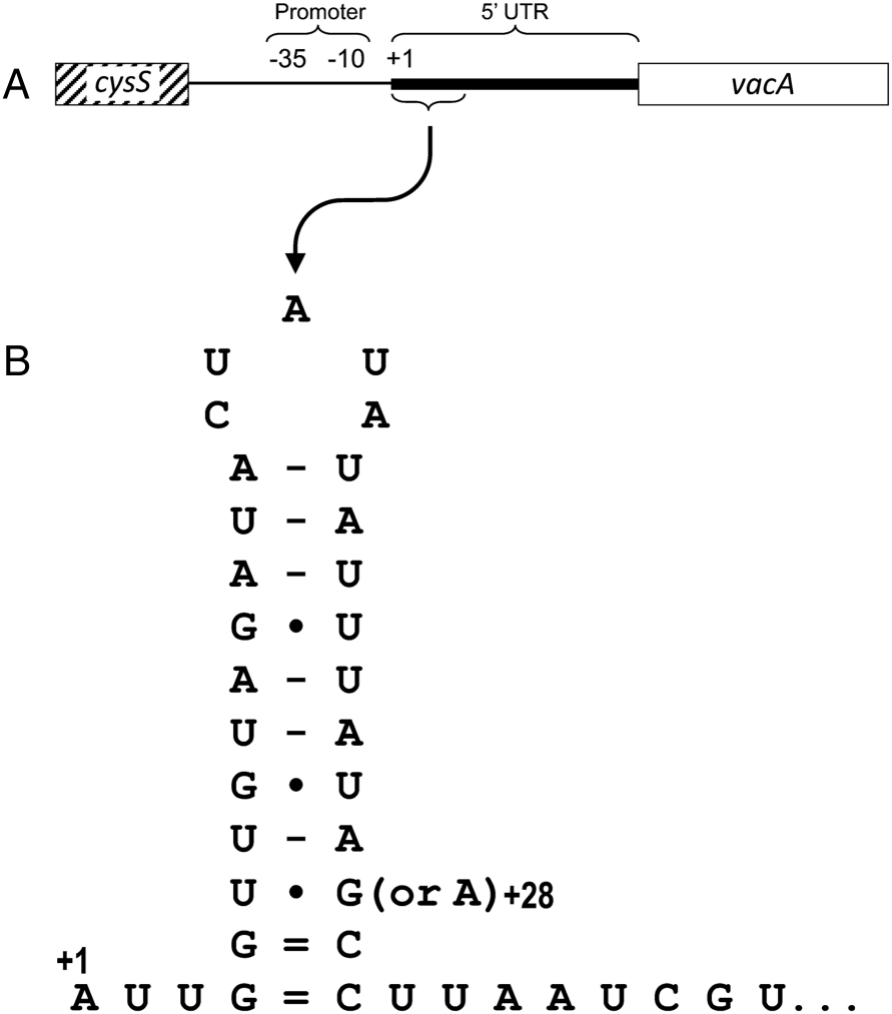
A predicted stem-loop sequence is present near the start of the *vacA* 5′ untranslated region (UTR). (A) The intergenic region between the 3′ end of the upstream gene *cysS* (diagonal hatched box) and the start of the *vacA* open reading frame (white box) is shown with the −35 and −10 consensus promoter sequence positions, the transcriptional start site (+1) and the 5′ UTR (thick line) indicated. (B) The predicted mRNA fold of a potential stem-loop sequence located at +4 to +30 in the *vacA* 5′ UTR, with a G/A polymorphism at position +28 shown.

**Figure 4:**
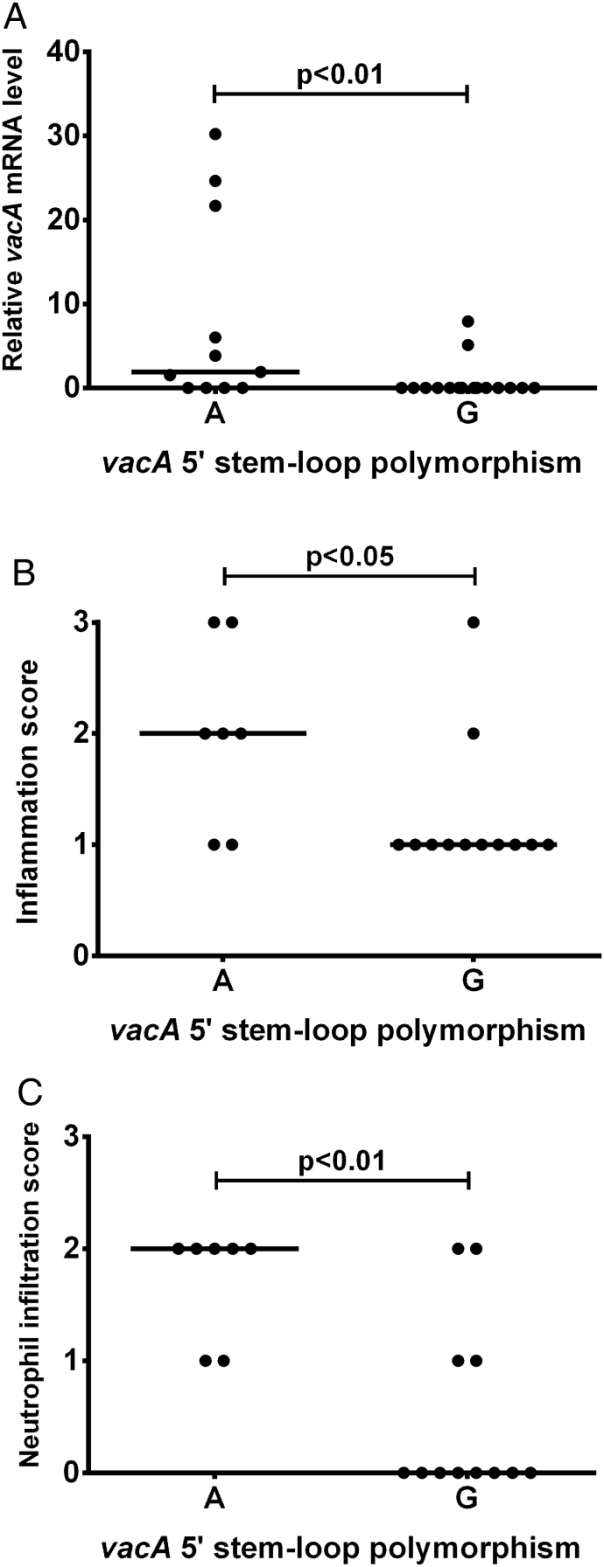
A natural G/A polymorphism within a potential stem-loop in the *vacA* 5′ untranslated region (UTR) is associated with in vivo *vacA* mRNA level, inflammation score and neutrophil infiltration. The nucleotide at position +28 in the *vacA* 5′ UTR (A or G) was determined by sequencing for the infecting strain from 27 patients. Gastric biopsies were used to quantify the relative in vivo *vacA* mRNA level by reverse transcriptase quantitative PCRs for all 27 patients (A), and scored for inflammation (B) and neutrophil infiltration (C) using the Sydney system for 19 patients infected with i1-type strains. Data were assessed by Mann–Whitney U tests for statistical significance.

We recently described a second inverted repeat located between +4 and +30 in the *vacA* 5′ UTR, predicted to form a stem-loop structure at the 5′ end of the transcript (figure 3).^24^ We showed that this potential stem-loop is important for stabilising *vacA* mRNA: disrupting its formation led to reduced mRNA half-life and decreased steady-state *vacA* transcript levels.^24^ At the third pairing position from the base, the 5′ stem is consistently thymine (uracil in mRNA) but the 3′ pairing nucleotide (position +28) can be either guanine (G(+28)) or adenine (A(+28)), the latter potentially stabilising the stem-loop more efficiently. In our previous work, we showed that substituting adenine for guanine at position +28 significantly increased *vacA* mRNA steady-state levels in two different strain backgrounds.^24^ Here we report that strains possessing A(+28) expressed more *vacA* in gastric biopsies than those with G(+28) (p<0.01; figure 4A). Furthermore, i1-type strains with A(+28) were associated with more inflammation (p<0.05; figure 4B) and neutrophil infiltration (p<0.01; figure 4C) than those with G(+28).

## Discussion

The vacuolating cytotoxin is a major virulence determinant of *H. pylori*. Previously, we described *vacA* polymorphisms that affect its toxicity and devised simple PCR-based typing systems to identify these alleles among *H. pylori* strains.^9–11^ ^22^ Subsequent *vacA* typing has shown that specific alleles, such as s1- and i1-types, are robust markers of increased disease risk in many Western populations.^6^ Here we show for the first time that in addition to differences in VacA toxicity between strains, *vacA* expression during *H. pylori* infection of the human stomach varies widely and is associated with the amount of inflammation and atrophy present. Previous studies have shown *vacA* expression varies widely between strains, but no correlation with promoter region polymorphisms was observed: sequence data were only reported for two strains studied by Forsyth *et al*,^16^ and there was insufficient variation at the +28 position in the 5′ UTR of the strains studied by Ayala *et al*^17^ to show the same association with *vacA* expression observed among our strains. An important difference in our study is that *vacA* mRNA levels were measured within gastric biopsies from patients with the infection, rather than from in vitro cultures of isolated strains.

Despite many polymorphisms in the *cysS*–*vacA* intergenic region, we found only one that associated with gastric *vacA* mRNA expression, located at position +28 in the 5′ UTR. However, we cannot rule out that multiple polymorphisms affect *vacA* expression and have confounding effects on each other, masking any association with our expression data. Candidate polymorphisms are those located at −32, −14 and −7 within the promoter consensus sites. Indeed, replacing guanine with thymine at position −14 reduces *vacA* transcription twofold;^23^ so it is possible that the G/A polymorphism we observed at this position would affect *vacA* mRNA levels when assessed in an isogenic system.

It was interesting that the *vacA* 5′ UTR was significantly more conserved than the upstream nucleotide sequence containing the promoter sites. One explanation could be sequence constraints imposed by important secondary structure formation in this untranslated mRNA region. Indeed, we have recently identified a potential stem-loop structure from +4 to +30 in the *vacA* 5′ UTR which we showed was important for stabilising the *vacA* mRNA transcript.^24^ Interestingly, the only polymorphism significantly associated with gastric *vacA* mRNA level and inflammation, G/A(+28), was located within this stem-loop sequence. In our previous work, site-directed mutagenesis was used to replace guanine with adenine at +28 in the *vacA* 5′ UTR of strains 60190 and SS1, allowing more favourable pairing with uracil at position +6 and potentially increasing the stability of the stem-loop. We found that isogenic mutants with A(+28) had significantly higher steady-state levels of *vacA* mRNA than their wild-type controls with G(+28).^24^ This supports our current finding that clinical isolates with adenine at +28 are associated with increased *vacA* mRNA level in gastric biopsies from patients infected with *H. pylori*.

A recent study found *vacA* expression to be higher in patients with gastric cancer than in those with non-atrophic gastritis or duodenal ulcer.^25^ This agrees with our finding that higher *vacA* expression in the gastric mucosa is associated with increased inflammation and the presence of atrophy. Therefore, the disease risk of a patient with *H. pylori* infection is affected by both the VacA type of the infecting strain and the level of *vacA* expression during infection. This is important because the association between more active s1m1- and more recently i1-type strains and PUD or GC is not absolute. Furthermore, in countries where most *H. pylori* strains are s1i1-type, these markers of VacA toxicity do not allow differential determination of disease risk. Our finding that a natural polymorphism at nucleotide +28 of the *vacA* transcript affects *vacA* mRNA level shows that specific naturally occurring polymorphisms may affect *vacA* expression. This and other polymorphisms affecting *vacA* transcript levels may provide important additional markers for determining which patients are at greatest risk of developing severe gastric or duodenal disease, particularly among those infected with more toxic *vacA* i1-type *H. pylori* strains.

**Take home messages**

- Expression of the *Helicobacter pylori* vacuolating cytotoxin gene A (*vacA*) within the infected gastric mucosa varies considerably between strains.
- The level of *vacA* mRNA expressed in vivo correlates with gastric inflammation and premalignant pathology.
- A polymorphism at nucleotide +28 with the *vacA* 5′ untranslated region of the transcript is associated with gastric *vacA* expression level and inflammation, and may provide an additional marker of disease risk.
