# Supplementary material for: Helicobacter pylori vacA transcription is genetically-determined and stratifies the level of human gastric inflammation and atrophy
Source: J Clin Pathol. 2016 May 17;69(11):968–73. doi: 10.1136/jclinpath-2016-203641 (PMC5136723; doi:10.1136/jclinpath-2016-203641)
Supplement: Supplementary figure [file jclinpath-2016-203641supp.pdf]

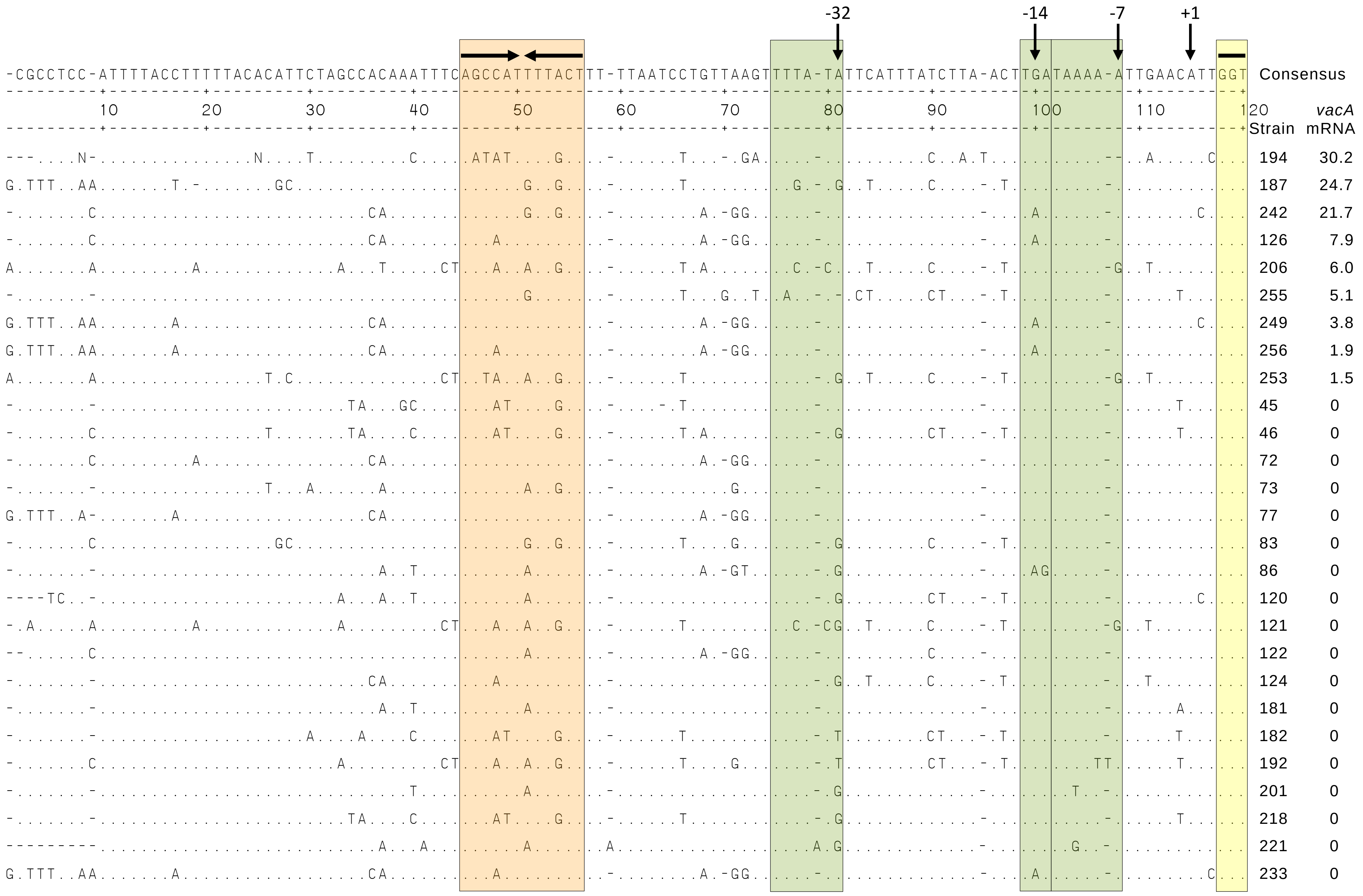
-32
-14
-7
+1
-CGCCTCC-ATTTTACCTTTTTACACATTCTAGCCACAAATTTCAGCCATTTTACTTT-TTAATCCTGTTAAGTTTTA-TATTCATTTATCTTA-ACTTGATAAAA-ATTGAACATTGGT
Consensus
10
20
30
40
50
60
70
80
90
100
110
120
Strain
vacA mRNA
194 30.2
187 24.7
242 21.7
126 7.9
206 6.0
255 5.1
249 3.8
256 1.9
253 1.5
45 0
46 0
72 0
73 0
77 0
83 0
86 0
120 0
121 0
122 0
124 0
181 0
182 0
192 0
201 0
218 0
221 0
233 0

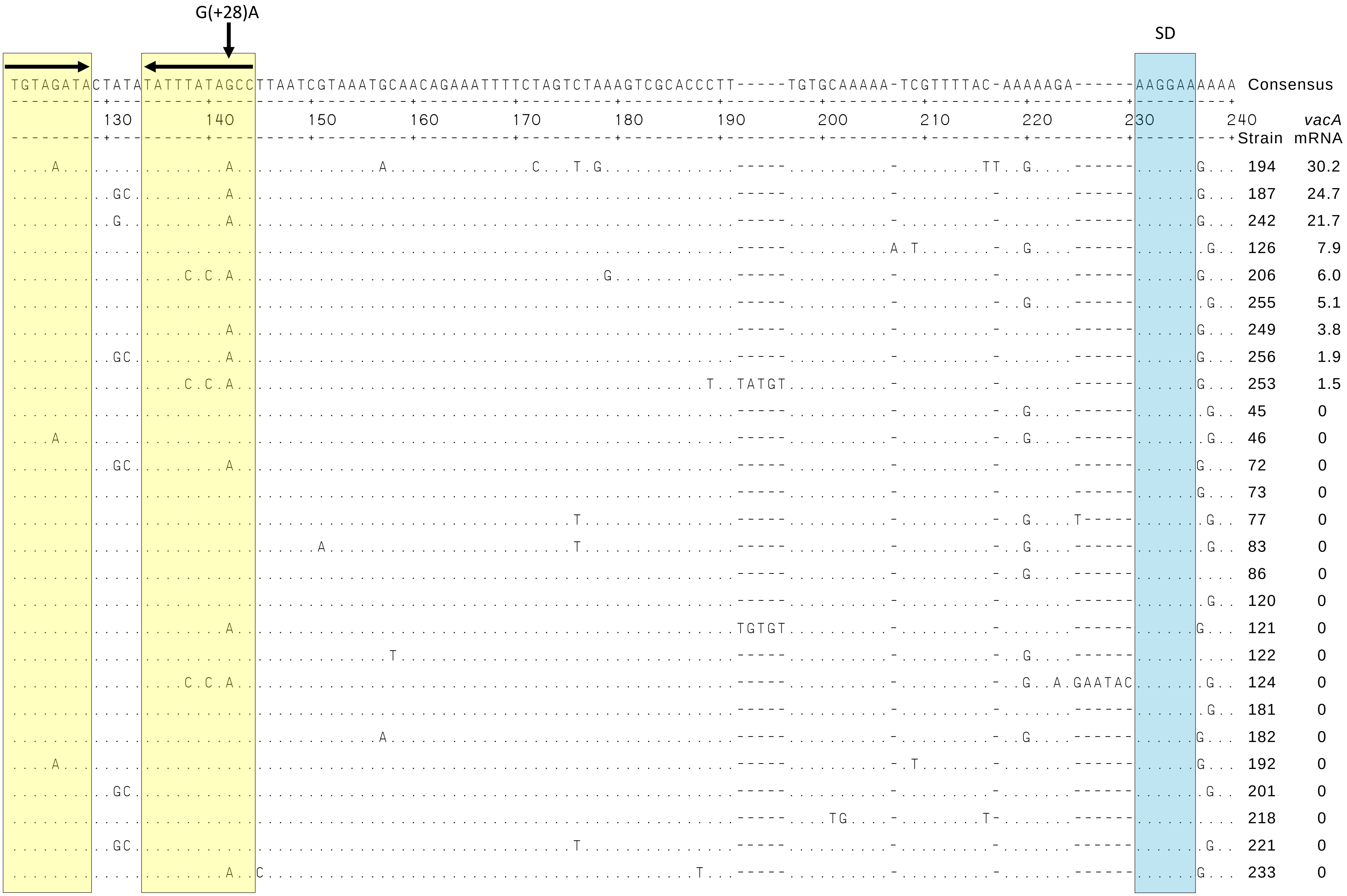

G(+28)A
SD
TGTAGATACTATATATTTATAGCCTTAATCGTAAATGCAACAGAAATTTTCTAGTCTAAAGTCGCACCCTT-----TGTGCAAAAA-TCGTTTTAC-AAAAAGA------AAGGAAAAAA Consensus
130 140 150 160 170 180 190 200 210 220 230 240
Strain
vacA mRNA
194 30.2
187 24.7
242 21.7
126 7.9
206 6.0
255 5.1
249 3.8
256 1.9
253 1.5
45 0
46 0
72 0
73 0
77 0
83 0
86 0
120 0
121 0
122 0
124 0
181 0
182 0
192 0
201 0
218 0
221 0
233 0

Supplementary Figure 1. Sequence alignment of the *cysS-vacA* intergenic region of 27 *H. pylori* clinical isolates ranked in order of in vivo relative *vacA* mRNA level from highest (top) to lowest (bottom). Relative in vivo *vacA* mRNA level (shown in the right-hand column) was quantified by reverse transcriptase quantitative polymerase chain reaction (RT-qPCR) using cDNA synthesised from total RNA purified from patient gastric biopsies. *H. pylori 16S rRNA* was used as a reference gene, and *vacA* mRNA level was measured relative to that of a comparator biopsy cDNA sample included in each run (assigned an arbitrary value of 100). The following features described in the Results section are annotated: an upstream inverted repeat (orange shading); the -35 and -10 promoter sequences (green shading); the 5' untranslated region stem loop (yellow shading); and the Shine-Dalgarno sequence (blue shading). The start of the *vacA* transcript is labelled +1, and the polymorphisms at nucleotides -32, -14, -7 and G(+28)A, described in the Results section, are indicated by arrows. Nucleotide identity to the consensus sequence is shown by a dot, and gaps in the alignment are indicated by a dash.
